# Cardiovascular fitness is associated with child adiposity at 5 years of age: findings from the ROLO longitudinal birth cohort study

**DOI:** 10.1186/s12887-023-04157-0

**Published:** 2023-07-08

**Authors:** Aisling A. Geraghty, Eileen C. O’Brien, Sophie Callanan, John Mehegan, Fionnuala M. McAuliffe

**Affiliations:** 1 UCD Perinatal Research Centre, School of Medicine, University College Dublin, National Maternity Hospital, Dublin, Ireland; 2 UCD School of Public Health, Physiotherapy and Sports Science, University College Dublin, Belfield, Dublin 4, Ireland

**Keywords:** Childhood obesity, Heart rate recovery, Step test, Cardiovascular health, Fitness

## Abstract

**Background:**

Cardiovascular fitness is strongly linked with metabolic risk; however, research is limited in preschool children. Although there is currently no simple validated measure of fitness in preschool children, heart rate recovery has been highlighted as an easily accessible and non-invasive predictor of cardiovascular risk in school-aged children and adolescents. We aimed to investigate whether heart rate recovery was associated with adiposity and blood pressure in 5-year-olds.

**Study design:**

This is a secondary analysis of 272 5-year-olds from the ROLO (Randomised cOntrol trial of LOw glycaemic index diet in pregnancy to prevent recurrence of macrosomia) Kids study. Three-minute step tests were completed by 272 participants to determine heart rate recovery duration. Body mass index (BMI), circumferences, skinfold thickness, heart rate, and blood pressure were collected. Independent *t*-tests, Mann-Whitney U, and Chi-square tests were used to compare participants. Linear regression models examined associations between heart rate recovery and child adiposity. Confounders included child sex, age at study visit, breastfeeding, and perceived effort in the step test.

**Results:**

The median (IQR) age at the study visit was 5.13 (0.16) years. 16.2% (n = 44) had overweight and 4.4% (n = 12) had obesity based on their BMI centile. Boys had a quicker mean (SD) heart rate recovery after the step test than girls (112.5 (47.7) seconds vs. 128.8 (62.5) seconds, *p =* 0.02). Participants with a slower recovery time (> 105 s) had higher median (IQR) sum of skinfolds (35.5 (11.8) mm vs. 34.0 (10.0) mm, *p* = 0.02) and median (IQR) sum of subscapular and triceps skinfold (15.6 (4.4) mm vs. 14.4 (4.0) mm, *p* = 0.02) compared to participants with a quicker recovery time. After adjusting for confounders (child sex, age at study visit, breastfeeding, effort in the step test), linear regression analyses revealed heart rate recovery time after stepping was positively associated with sum of skinfolds (B = 0.034, 95% CI: 0.01, 0.06, *p* = 0.007).

**Conclusion:**

Child adiposity was positively associated with heart rate recovery time after the step test. A simple stepping test could be used as a non-invasive and inexpensive fitness tool in 5-year-olds. Additional research is needed to validate the ROLO Kids step test in preschool children.

## Introduction

In 2020, approximately 38.2 million children under 5 years of age had overweight or obesity worldwide [1]. Although the global prevalence of overweight or obesity is lower in children than in adults, the rate of increase in children is higher [2]. The lifetime costs of childhood obesity in Ireland were recently estimated to be €4.6 billion, representing a massive economic burden [3]. With one in five Irish children currently having overweight or obesity, the risk of raised blood pressure, dyslipidaemia, and insulin resistance increases from an early age [4, 5].

Cardiovascular fitness is one indicator that is closely related to metabolic health and adiposity in adults and school-aged children [6]. Children’s cardiovascular fitness has sharply declined over previous decades worldwide; however, recent statistics suggest that the rate of decline has plateaued [7–9]. In 2020, the American Heart Association recognised cardiovascular fitness as a key health predictor that should be routinely monitored in clinical practice for all school-aged youth [10]. Evidence suggests that the development of cardiovascular fitness levels begins from a young age and tracks into adulthood to predict future health profiles [11].

Strong links have been identified in school-aged children and youth between higher fitness and lower adiposity and reduced cardiometabolic risk [12]. Additional research has highlighted the inverse relationship between physical fitness in children and cardiometabolic risk factors [13]. Less is known about the relationship between cardiovascular fitness and health indicators in preschool ages. Despite this, it is reasonable to consider cardiovascular fitness is also an important marker of health in early childhood based on evidence in older populations. Thus, additional investigation of cardiovascular fitness in preschool years is necessary to increase our understanding of its role in early life.

At present, there are no validated, submaximal field tests to identify preschool children with low cardiovascular fitness levels who would benefit from early intervention. Heart rate recovery time duration has been highlighted as an accurate indicator of cardiovascular fitness level in children and adolescents [14]. It is the difference between the peak heart rate during exercise and the heart rate at a specific time interval following the onset of recovery [15]. Heart rate recovery time duration is an easily accessible proxy for evaluating cardiovascular fitness that is suitable for preschool children due to its non-invasive nature [14].

Delayed heart rate recovery is associated with cardiovascular disease and all-cause mortality in adults [15]. Therefore, it is of clinical interest to determine whether this relationship is consistent in younger populations. Several studies in children and adolescents have reported inverse associations between heart rate recovery and obesity traits and metabolic risk factors [16–18]. Investigations exploring the link between heart rate recovery and health markers in preschool children are limited and more research in this area is warranted.

We aimed to address the paucity of information regarding the influence of cardiovascular fitness on adiposity and blood pressure in 5-year-olds by investigating these factors in Irish children from the ROLO (Randomised cOntrol trial of LOw glycaemic index diet in pregnancy to prevent recurrence of macrosomia) Kids cohort. The case for this investigation is important, given the children of the ROLO study are considered at higher risk for excess adiposity given the mothers of the participants had a prior history of a macrosomic pregnancy [19]. Simple three-minute stepping tests have been highlighted as innovative methods of estimating heart rate recovery in youth [14, 20, 21]. We developed a modified, simple “ROLO Kids step test” as a suitable method of estimating heart rate recovery in 5-year-old children. We hypothesised that a slower heart rate recovery would be related to higher child adiposity and blood pressure at 5-years of age.

## Methods

### Study design and population

This is a secondary analysis of 272 children from the ROLO Kids Study, a longitudinal follow-up of children born into the randomised control trial, ROLO study in Dublin, Ireland [19]. Healthy mothers with a history of previous macrosomic delivery (birthweight > 4Kg) were initially recruited into the ROLO study during their second pregnancy in the National Maternity Hospital, Dublin, between 2007 and 2011. Women were randomly assigned into the intervention or control arm; the intervention group received low glycaemic index dietary advice from early pregnancy and the control group received routine antenatal care [19]. No differences in birthweight were noted from the intervention, but several maternal benefits were observed including less gestational weight gain, improved insulin resistance, and better dietary intakes [19, 22]. The ROLO study has become a longitudinal birth cohort with follow-ups at multiple timepoints in childhood [22–25]. Informed written maternal consent was obtained at each follow up visit and verbal assent was obtained from the child participants. The Current Controlled Trials registration number for the ROLO study was ISRCTN54392969, first registered on 10/08/2009 [26].

### ROLO kids study visit

Eligibility to be included in this analysis required participation in the ROLO pregnancy study and attendance at a ROLO Kids study visit at 5-years of age. Once the child became eligible, the research team contacted the mother of the study child and invited them to attend a study visit with the research team. At the study visit, the mother of the study child provided informed, written consent and verbal assent was obtained from their child. Of 403 children who returned at 5-years of age, only children who completed a step test were included in this analysis. Data available for each parameter in this analysis varied depending on the co-operation of the child and data collection procedures at the time of the study visit. Mothers completed demographic, health, and lifestyle questionnaires.

### Exposure

#### ROLO kids step test

The ROLO Kids step test was based on the protocol published by Jankowski et al. which used the 3-minute Kasch Pulse Recovery test (KPR test) [21]. The KPR test used a metronome among children aged 6–12 years. Based on experience of conducting research with 5-year-old children, the researchers agreed the KPR test had to be modified to suit a preschool age cohort. The ROLO Kids step test was modified to omit stepping to the beat of a metronome, as it was not considered feasible to use among preschool children. Using a 25 cm (9.8 inches) step, the children were instructed to step up and down, one foot at a time, as many times as possible for 3 min. Before stepping commenced, a pedometer was attached to the child’s clothing at the hip level to record number of steps. Children were instructed to step as quickly as possible in a consistent manner over 3 min and gamification strategies were employed with the researcher stepping simultaneously with the child. Children were encouraged to maintain the same, self-directed stepping pace throughout. The researchers assessed the overall perceived effort of the child according to a 7-point scale shown in Table 1. Two researchers who witnessed the step test independently assessed the perceived effort of the child to reduce potential bias and averages of both scores were used. Perceived effort was categorised into “good effort” (rating 1–4) or “poor effort” (rating 5–7). All children who completed the step test were analysed, regardless of perceived effort rating.

**Table 1:** Perceived effort 7-point scale for ROLO Kids Step Test

E	EXCELLENT	1
VG	VERY GOOD	2
G	GOOD	3
FG	FAIR TO GOOD	4
F	FAIR	5
PF	POOR TO FAIR	6
P	POOR	7

### Outcomes

#### Cardiovascular fitness

Heart rate recovery time duration was used to provide an indicator of cardiovascular fitness level in the child based on previous research [14]. The child was seated at rest for at least 5 min and baseline heartrate was measured using a finger pulse oximeter (CMS-50QA Pediatric Finger Pulse Oximeter). Immediately after finishing 3 min of stepping, the child was asked to sit down and their heart rate was measured. Measurements were repeated every 30 s until heart rate returned to baseline and recovery time duration was noted.

#### Anthropometry

A trained research nutritionist/dietitian obtained the child anthropometric measurements at the study visit. Weight was recorded (in light clothing) to the nearest 0.1 kg using a SECA (SECA gmbh & co. Kg. Germany) scales and height was measured using a wall mounted stadiometer to the nearest 0.1 centimetre. Body mass index (BMI) was then calculated (kg/m^2^). Weight and BMI values were converted to standardised scores (BMI-SDS) and centiles relative to 1990 UK reference data using the Excel LMS Growth macro [27, 28]. BMI centiles were categorised as having a normal weight (between >-2 and < 1 SD), having overweight, (> 1 SD) or having obesity (> 2 SD) according to World Health Organization cut-off points [29]. Abdominal circumference was measured to the nearest 0.1 centimetre using a SECA (SECA gmbh & co. Kg. Germany) measuring tape. Skinfold thicknesses (bicep, triceps, subscapular, and thigh) were measured to the nearest 0.1 mm using a Holtain Tanner/Whitehouse caliper (Holtain Ltd, Crymych, UK). Waist-to-height ratio, sum of skinfolds, sum of subscapular and triceps skinfold, and subscapular-to-triceps skinfold ratio were calculated as proxy measures of adiposity. Previous research suggests that using multiple skinfold sites (and combining measures) are superior to the measurement of one site in accurately assessing adiposity in children [30].

#### Cardiovascular health

Cardiovascular health was defined as the assessment of systolic blood pressure, diastolic blood pressure, and heart rate at rest. These measures were chosen to provide a non-invasive insight into cardiovascular health that are simple and quick to obtain. A trained researcher measured heart rate, systolic blood pressure, and diastolic blood pressure at rest using the validated electronic sphygmomanometer Omron blood pressure monitor (Omron M6 Comfort oscillometric device HEM-7321-E, Omrom Healthcare, Kyoto, Japan). Participants were seated with limbs uncrossed with feet firmly on the ground and the arm supported at the heart level on a nearby even surface. Blood pressure and heart rate measures were obtained after at least 5 min of rest in the sitting position. Children were asked to remain still by the researcher for the duration of the assessment for an accurate reading. Systolic and diastolic blood pressure centiles were calculated using the LMS method for each participant according to age-, sex- and height-specific reference data published by Flynn et al. [31].

### Statistical analyses

Statistical analyses were performed using the IBM Statistical Package for the Social Sciences version 24.0 (SPSS Inc, Chicago, IL, USA). All variables were assessed for normality by visual inspection of histograms. Relationships between the central tendencies were examined using student T-tests, for normally-distributed data, or Mann Whitney-U tests for non-normal data. Heart rate recovery data was stratified according to the median (“Slow Heart Rate Recovery” >105 s and “Quick Heart Rate Recovery” <105 s). Differences between groups were explored using student T-tests, Mann Whitney-U tests or Chi-square tests as appropriate. Pearson correlation was used for normally distributed data, with Spearman’s correlation for non-normally distributed data to measure the correlation between heart rate recovery after the step test with adiposity and cardiovascular measures. Significant correlations were used to create multiple regression models using a forced entry approach to control for confounders which were selected a priori (effort in the step test, child sex, age at study visit, and exposure to breastfeeding). Perceived effort and age at appointment were controlled for to account for differences within the sample population. Models were adjusted for breastfeeding exposure and child sex because previous literature suggests they may influence child adiposity in preschool ages [32, 33]. Statistical significance for all analyses was defined as *p* < 0.05.

## Results

### Cohort characteristics from the ROLO kids study

Cohort characteristics are displayed in Table 2. This analysis included 272 children who participated in the ROLO Kids study visit, 45.5% of which were male (n = 124). At the study visit, the median (IQR) age of participants was 5.13 (0.16) years and boys were slightly younger at the study visit than girls (5.11 (0.16) years vs. 5.14 (0.17), *p* = 0.045). The median (IQR) BMI was 16.0 (1.7) kg/m^2^. According to their BMI centile classification, 16.2% (n = 44) of children had overweight and 4.4% (n = 12) of children had obesity. Significant mean differences (SED) were found for birthweight (0.16 (0.05) kg, *p* = 0.003), height (1.22 (0.52) cm, *p* = 0.02) and chest circumference (0.79 (0.32) cm, *p* = 0.015) between boys and girls. Compared to girls, boys had significantly lower median (IQR) waist-to-height ratio (0.48 (0.04) vs. 0.49 (0.01), *p* = 0.026), sum of skinfolds (33.4 (10.9) mm vs. 36.85 (11.0) mm, *p* < 0.001) and sum of subscapular and triceps skinfold (14.2 (4.2) mm vs. 16.2 (4.3) mm, *p* < 0.001), indicating lower adiposity. There were no other significant differences in measures of anthropometry or cardiovascular health between boys and girls.

**Table 2:** Characteristics of the 5-year-old children in the ROLO cohort

		Total		Boys			Girls				
	n	Mean/Median	SD/ IQR	n	Mean/Median	SD/ IQR	n	Mean/Median	SD/ IQR	*P*	
RCT group (Intervention), n (%)^+^	272	142 (52.2)		124	64 (51.6)		148	78 (52.7)		0.858	
Birthweight (kg)	272	4.03	0.45	124	4.12	0.68	148	3.95	0.4	**0.003**	
Birthweight centile*	248	79.8	37.4	113	80.5	35.8	135	77.1	38.2	0.3	
Smoked during pregnancy, n (%)^+^	272	7 (2.6)		124	2 (1.6)		148	5 (3.4)		0.36	
Breastfed, n (%)^+^	242	163 (59.9)		111	71 (57.3)		131	91 (62.2)		0.3	
***Anthropometry***											
Age at follow up (years)*	272	5.13	0.16	124	5.11	0.16	148	5.14	0.17	**0.045**	
Weight (kg)*	272	19.8	3.2	124	19.95	2.9	148	19.6	3.4	0.18	
Weight centile*	272	65.5	41.0	124	65.0	39.0	148	65.5	42.0	0.48	
Height (cm)	272	111.52	4.39	124	112.18	4.0	148	110.96	4.63	**0.02**	
Height centile*	272	60.5	47.0	124	62.50	39.0	148	58.5	57.0	0.27	
BMI (kg/m^2^)*	272	16.04	1.68	124	16.09	1.18	148	16.16	1.34	0.65	
BMI centile*	272	65.5	42.0	124	63.5	44.0	148	66.0	40.0	0.73	
Overweight, n (%)^a+^	272	44 (16.2)		124	21 (16.9)		148	23 (15.5)		0.612	
Obesity, n (%)^a+^	272	12 (4.4)		124	7 (5.6)		148	5 (3.4)		-	
Chest circumference (cm)	271	56.33	2.7	124	56.76	2.51	147	55.96	2.81	**0.015**	
Abdominal circumference (cm)*	272	54.6	5.1	124	54.35	4.5	148	54.75	5.6	0.34	
Waist-to-height ratio*	272	0.48	0.04	124	0.48	0.04	148	0.49	0.05	**0.026**	
Sum of skinfolds (mm)*	251	35.4	11.6	117	33.4	10.95	134	36.85	11.05	**< 0.001**	
Subscap&triceps skinfold (mm)*	256	15.2	4.35	119	14.2	4.2	137	16.2	4.3	**< 0.001**	
Subscapular-to-triceps ratio*	256	0.6	0.15	119	0.6	0.12	137	0.6	0.17	0.98	
***Cardiovascular Health***											
Heart rate (bpm)	261	92.27	11.78	119	91.87	10.94	142	92.61	12.47	0.61	
Systolic blood pressure (mmHg)	247	99.16	9.41	116	100.05	9.05	131	98.37	9.69	0.16	
Systolic blood pressure centile*	247	73.0	38.0	116	74.0	40.0	131	72.0	36.0	0.32	
Diastolic blood pressure (mmHg)*	247	60.0	10.0	116	59.0	10.0	131	61.0	11.0	0.32	
Diastolic blood pressure centile*	247	73.0	35.0	116	71.0	36.0	131	74.0	34.0	0.84	

Normally distributed data is reported as mean (standard deviation) unless otherwise stated. *Non-normal data is reported as median (interquartile range). ^+^Categorical data is reported as n (%). ROLO: Randomised cOntrol trial of Low glycaemic index diet in pregnancy to prevent recurrence of macrosomia, RCT: Randomised Controlled Trial, BMI: Body Mass Index. Statistical comparisons by student T-test, Mann-Whitney U or Chi-square tests. ^a^BMI centiles categorised according to World Health Organisation cut-offs for children aged 5 to 19-years. Significant at *P* < 0.05

### Results of the ROLO kids step test

The results of the ROLO Kids step test are shown in Table 3. A total of 272 children completed the ROLO Kids step test. Data available across some parameters vary in cases where researchers were unable to collect finishing heart rate, heart rate increase, or heart rate recovery due to a lack of co-operation from participants. In addition, perceived effort and the number of steps taken were added as parameters of interest at a later time-point during the data collection period and so was not collected for all participants. Total numbers available for each parameter are reported in Table 3. The average (SD) number of steps taken by participants in the step test was 275 (49.9). 91.9% (n = 180) had a “good” perceived effort rating in the test. Heart rate increased by an average (SD) 41.9 (22.4) bpm during the step test. When stratified by sex, there was a significant mean difference (SED) in heart rate recovery after the step test (-16.3 (7.0) seconds, *p =* 0.02) between boys and girls. Boys and girls had a similar starting heart rate (mean difference − 0.85, SED 1.6 bpm), end point heart rate (mean difference − 4.6, SED 2.9 bpm), and heart rate increase (mean difference − 4.0, SED 2.8 bpm). The number of steps taken by boys and girls were similar (mean difference 3.53, SED 7.95 steps). The number of children who received a perceived effort rating of “good” was similar between boys and girls (91.5% vs. 92.2%).

**Table 3:** ROLO Kids Step Test Results of the 5-year-old children in the ROLO cohort

		Total		Boys			Girls				
	n	Mean (SD)		n	Mean (SD)		n	Mean (SD)		*P*	
Starting heart rate	272	97.18	13.07	124	96.72	12.09	148	97.57	13.86	0.59	
Finishing heart rate	263	139.35	23.12	120	136.85	22.25	143	141.44	23.7	0.11	
Heart rate increase	263	41.86	22.43	120	39.82	21.63	143	43.57	23.02	0.18	
Heart rate recovery (seconds)	247	121.18	56.56	115	112.47	47.67	132	128.77	62.49	**0.02**	
Perceived effort (“good”), n (%)^+^	196	180 (91.8)		94	86 (91.5)		102	94 (92.2)		0.87	
Steps taken	163	275.6	49.94	68	277.66	44.44	95	274.13	53.72	0.66	

Normally distributed data is reported as mean (standard deviation) unless otherwise stated. ^+^Categorical data is reported as n (%). ROLO: Randomised cOntrol trial of Low glycaemic index diet in pregnancy to prevent recurrence of macrosomia. Statistical comparisons by student T-test or Chi-square tests. *Significant at *P* < 0.05

### Differences in child anthropometry and cardiovascular health based on heart rate recovery time after the ROLO kids step test

Heart rate recovery data was stratified according to the median (“Slow Heart Rate Recovery” >105 s and “Quick Heart Rate Recovery” <105 s). In Table 4, analysis using Chi-square Bonferroni post hoc test revealed more participants with a slower recovery time (> 105 s) were classified as having perceived effort rating of “good” (96.1% vs. 88%, *p* = 0.04), compared to participants with a quicker recovery time. Participants with a slower recovery time (> 105 s) higher median (IQR) sum of skinfolds (35.5 (11.8) mm vs. 34.0 (10.0) mm, *p* = 0.02) and median (IQR) sum of subscapular and triceps skinfold (15.6 (4.4) mm vs. 14.4 (4.0) mm, *p* = 0.02) compared to participants with a quicker recovery time. There were no significant differences between the slower and quicker heart rate recovery groups in other measures of anthropometry or cardiovascular health.

**Table 4:** Comparison between participants with slow and quick levels of heart rate recovery time

	Slow Heart Rate Recovery (> 105 s)			Quick Heart Rate Recovery (< 105 s)			
	n	Mean/ Median	SD/IQR	n	Mean/ Median	SD/IQR	*P*
***ROLO Kids Step Test***							
Steps taken	83	277.0	51.0	68	288.5	62.0	0.32
Perceived effort (“good”), n (%)^+^	102	98 (96.1)		75	66 (88.0)		**0.04**
***Anthropometry***							
Weight (kg)	142	20.01	2.38	105	20.02	2.38	0.97
Weight centile*	141	66.00	38.00	105	64.00	45.00	0.82
Height (cm)	142	111.19	4.16	105	111.55	4.39	0.51
Height centile*	141	57.00	46.00	105	63.00	49.00	0.58
BMI (kg/m^2^)	142	16.15	1.29	105	16.05	1.22	0.57
BMI centile*	141	68.00	41.00	105	61.00	39.50	0.40
Overweight, n (%)^a+^	141	24 (16.9)		105	14 (13.3)		0.725
Obesity, n (%)^a+^	141	6 (4.2)		105	4 (3.8)		-
Chest circumference (cm)	141	56.21	2.62	105	56.14	2.61	0.82
Abdominal circumference (cm)	141	54.81	3.61	105	54.74	3.84	0.89
Waist-to-height ratio	141	0.49	0.03	105	0.49	0.03	0.94
Sum of skinfolds (mm)*	130	35.5	11.75	97	34.0	10.0	**0.02**
Subscap&triceps Sf (mm)*	131	15.60	4.40	101	14.40	4.00	**0.02**
Subscapular-to-triceps ratio*	131	0.60	0.13	101	0.60	0.20	0.78
***Cardiovascular Health***							
Heart rate (bpm)*	132	94.0	14.0	104	92.0	15.0	0.23
Systolic blood pressure (mmHg)	121	99.89	9.1	101	98.72	8.73	0.33
Systolic blood pressure centile*	121	74.0	37.0	101	73.0	35.0	0.24
Diastolic blood pressure (mmHg)	121	60.54	7.93	101	60.9	8.11	0.73
Diastolic blood pressure centile*	121	74.0	33.0	101	74.0	32.0	0.73

Normally distributed data is reported as mean (standard deviation) unless otherwise stated. *Non-normal data is reported as median (interquartile range). ^+^Categorical data is reported as n (%). ROLO: Randomised cOntrol trial of Low glycaemic index diet in pregnancy to prevent recurrence of macrosomia. Statistical comparisons by student T-test, Mann-Whitney U, or Chi-square tests. ^a^BMI centiles categorised according to World Health Organisation cut-offs for children aged 5 to 19-years. Significant at *P* < 0.05.

### Associations between heart rate recovery time after the ROLO kids step test and child adiposity

Pearson correlation tests were used to assess unadjusted associations between heart rate recovery with anthropometry, blood pressure, and heart rate at rest. Heart rate recovery after completing the step test was positively correlated with sum of skinfolds (r = 0.164, *p* = 0.01) and sum of subscapular and triceps skinfold (r = 0.138, *p* = 0.04), both of which are indicators of adiposity [30]. There were no significant correlations between heart rate recovery and blood pressure or heart rate at rest. Significant correlations were further investigated using linear regression models that were controlled for child sex, age at study visit, breastfeeding exposure, and perceived effort in the step test. Adjusted regression analyses revealed heart rate recovery remained significantly associated with the sum of skinfolds (B = 0.034, 95% CI 0.01, 0.06, *p* = 0.007). Each 1-SD (1 cm) increment in sum of skinfold thickness corresponded to 3.4 s of an increase in heart rate recovery time. The adjusted model is shown in Table 5.

**Table 5:** Linear regression model for sum of skinfold measures in the ROLO Kids participants

	B	*P*	CI Lower	CI Upper	r² adj	F	*P*	
Child Sex	4.483	0.002*	1.63	7.33	0.134	4.59	**0.001**	
Age at study visit (years)	7.848	0.14	-2.68	18.37				
Breastfed	1.136	0.45	-1.85	4.12				
ROLO Kids Step Test Effort (Good or Poor)	2.005	0.49	-3.76	7.77				
Heart Rate Recovery (seconds)	0.034	0.007*	0.01	0.06				

ROLO: Randomised cOntrol trial of Low glycaemic index diet in pregnancy to prevent recurrence of macrosomia. CI: Confidence interval, *Significant at ***P***  < 0.05. Model adjusted for child sex, age at study visit, breastfeeding exposure, and perceived effort in the step test.

## Discussion

We found that heart rate recovery after a simple stepping test was positively associated with adiposity in preschool children at 5-years of age. This study also observed significant sex differences in cardiovascular fitness, as boys had a faster heart rate recovery time after the step test than girls. No favourable associations were observed between heart rate recovery and cardiovascular health at rest.

Current research recognises the importance of examining fitness and metabolic health in adults due to the gradual manifestation of chronic disease [34]. A significant shift in focus towards earlier intervention is warranted by the rising prevalence of cardiovascular risk factors in childhood [35]. Research in children has shown that higher fitness levels have a substantially lower risk of overweight and obesity than those with lower fitness levels [34, 36]. Cardiovascular fitness has been identified as the key moderator of the association between physical activity and abdominal adiposity in school-aged children and adolescents [37]. Our novel analysis expands on the importance of assessing these factors in preschool ages, due to the high risk of excess adiposity in childhood tracking into adolescence and adulthood [38].

Heart rate recovery has been described as a simple, non-expensive method of estimating cardiovascular fitness, and its use in clinical studies in adults is well established [39, 40]. We found that heart rate recovery after a simple stepping test was positively associated with adiposity in preschool children at 5-years of age, as estimated using skinfold measures. This association remained significant after controlling for several confounding factors including child sex, age at study visit, breastfeeding exposure, and perceived effort in the step test. The step test had a high level of participation with 91.9% of children putting in a good effort and the sum of skinfolds was correlated with heart rate recovery regardless of effort put in by the child. Our findings suggest that the ROLO Kids step test may be a comprehensive estimate of fitness and risk of obesity in preschool ages.

While additional efforts are needed to validate the ROLO Kids step test, previous studies that used similar step tests reported consistent associations [20, 21, 41]. Compared to a 45-second squat test that required participants to complete 30 squats paced by a metronome, Bruggeman et al. reported that fitness scores from a three-minute step test correlated best with treadmill VO_2max_ test results in 10 to 17-year-olds [20]. Additional recent research found heart rate after a three-minute step test was positively correlated with BMI z-score, waist circumference z-score, and insulin resistance in 8 to 15-year-olds with overweight and obesity [42]. Likewise, Suriano et al. reported lower peak heart rate during a three-minute step test was associated with significantly reduced triglycerides, and lower fasting glucose, insulin, and insulin resistance amongst children with healthy weight aged 6 to 13-years [43]. Finally, 10 to 12-year-old children in the upper-quartile of heart rate recovery after a three-minute step test had an increased risk of dyslipidaemia compared to those in the lower quartile [14]. These findings indicate that a step test may be a useful exercise to help identify cardiovascular risk factors in children.

This study observed several sex differences in adiposity and cardiovascular fitness. At 5-years of age, boys were significantly taller, and leaner compared to girls, consistent with biological differences in metabolism from early life [33]. Previous research has shown that sex differences in adiposity have been evident in the ROLO cohort from infancy. Factors such as parental adiposity, maternal cytokines in utero, and the placental phenotype have been associated with differences in infant anthropometry between males and females [44–46]. This research highlights the potential need for sex-specific obesity prevention strategies for predisposed children in early childhood years. We also found males had a quicker heart rate recovery time than girls after the ROLO Kids step test. Simahee et al. found similar differences in 10-12-year-olds, where a higher percentage of boys were in the lower heart rate recovery quartile following a three-minute step test compared to a higher percentage of girls in the upper quartile [14]. Given that current literature supports the long-term impact of childhood fitness levels on cardiovascular risk factors in later life [47], greater focus on promoting adequate fitness levels in both sexes from preschool years is needed. Differences in heart rate recovery between boys and girls may also be influenced by biological factors such as aerobic fitness, autonomic regulation at rest, and resting heart rate from a young age [48, 49].

A secondary finding of this study is that the ROLO Kids step test may serve as a novel and innovative method of assessing cardiovascular fitness in preschool children. To our knowledge this is the first step-based method of assessing cardiovascular fitness in 5-year-old children. Three-minute step tests are considered simple and practical assessments that can be easily replicated with limited equipment, space and training requirements [42]. Often, it is not possible to have expensive equipment such as a treadmill, or the space needed to carry out assessments, like the 20-meter shuttle run, in small areas or health clinics or doctors’ offices for a routine health screening [20]. Furthermore, evidence suggests children often experience discomfort and pacing challenges during extensive fitness testing which may limit the reliability of results [48, 50]. The simple ROLO Kids step test could be used in a doctor’s office as a quick estimate of fitness level and associated risk in children. The non-invasive nature of the step test is particularly important for the age group of this cohort, which may play a role in reducing healthcare-related anxiety in preschool ages. Further research is needed to replicate our results in larger paediatric cohorts. Future validation of the ROLO Kids step test may promote its use to target preschool children who may benefit from early obesity preventative interventions.

This analysis had many strengths including the use of accurate objective anthropometry and body composition measurements that were collected by trained researchers. This analysis considered a wide range of variables and included valuable data related to early years which were used to provide additional context for the relationship between cardiovascular fitness and adiposity. The ROLO Kids step test is an innovative method to assess fitness in this age group and could easily be replicated in a small office space with low financial burden. It is important to acknowledge that the non-standardized approach to the use of the ROLO Kids step test and determination of heart rate recovery is a confounding factor. It is plausible that heart rate recovery levels were clouded by differences in effort due to free cadence. The assessment of perceived effort was used to control for this by including it as a confounder in the multivariate analysis. However, it should be acknowledged that the assessment of perceived effort by two researchers in this study was a subjective measurement. Another limitation may be the use of the 7-point scale to assess effort in this analysis. As the scale was not evenly weighted, it could potentially skew the results and result in very small numbers in the “poor effort” group. Future analyses may use a different scale that is equally weighted so that effort or participation with the step test could be better assessed. The interpretation of our findings from the ROLO Kids step test are limited by a lack of validation. The results of this study should encourage efforts to build stronger evidence to determine the validity of the ROLO Kids step test in larger paediatric cohorts.

## Conclusion

Child adiposity was positively associated with heart rate recovery time after completing an exercise step test. With heart rate recovery being a proxy for cardiovascular fitness, the ROLO Kids step test could be used as a submaximal, field measure of fitness in 5-year-old children in research and clinical settings. Further research and validation of these findings is required to expand on the importance of cardiovascular fitness in preschool children.

## Data Availability

The datasets used and/or analysed during the current study are available from the corresponding author on reasonable request.

## Abbreviations

ROLO: Randomised cOntrol trial of LOw glycaemic index diet in pregnancy to prevent recurrence of macrosomia
BMI: Body Mass Index
